# CRC-Former: frequency-domain adaptive swin-transformer for colorectal cancer histopathology classification

**DOI:** 10.3389/fphys.2026.1792357

**Published:** 2026-02-24

**Authors:** Lei Chen, Chenguang Li, Fanqi Meng, Jiandong Tai, Kun Wang

**Affiliations:** 1 Department of Colorectal and Anal Surgery, General Surgery Center, The First Hospital of Jilin University, Jilin, China; 2 Department of Ophthalmology, The First Hospital of Jilin University, Jilin, China

**Keywords:** colorectal cancer, haar wavelet transform, histopathology image classification, state-space model, swin-transformer

## Abstract

**Introduction:**

Colorectal cancer (CRC) diagnosis from whole-slide histopathology images remains challenging due to pronounced tissue heterogeneity, multi-scale morphological variations, and the subtle nature of early neoplastic changes. While deep learning models have shown promise, conventional architectures struggle to simultaneously capture fine-grained texture cues and global architectural context, often overlooking diagnostically critical frequency-domain signatures.

**Methods:**

To address these limitations, we propose CRC-Former, a novel hybrid architecture that synergistically integrates frequency-aware representation learning with efficient cross-scale sequence modeling. Specifically, CRC-Former introduces two key components: (i) a Frequency-aware Global-Local Transformer Block (FGT), which decomposes features via Haar wavelet transform and applies orientation-specific sliding-window attention in distinct subbands to enhance sensitivity to multi-directional pathological textures; and (ii) a Cross-Scale Mamba Block (CSM), which leverages selective state-space modeling to fuse hierarchical features across resolutions with linear complexity.

**Results:**

Evaluated on the large-scale Chaoyang CRC dataset, CRC-Former achieves state-of-the-art performance, outperforming strong baselines.

**Discussion:**

Our work demonstrates that explicit integration of signal processing priors with modern sequence modeling offers a powerful paradigm for robust, interpretable, and scalable computational pathology.

## 1 Introduction

**FIGURE 1:**
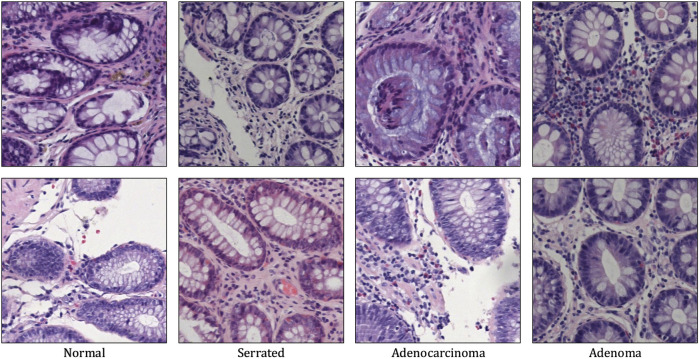
Representative H&E-stained histopathology patches from the four diagnostic categories in the Chaoyang dataset, representing (from left to right): normal mucosa, serrated lesion, adenocarcinoma, and adenoma. The increasing architectural and cytological atypia illustrate the challenges in automated CRC classification.

Colorectal cancer (CRC) ranks as the third most prevalent malignancy and a leading cause of cancer-related death globally, with early detection playing a decisive role in therapeutic planning and long-term survival Anusha and Reddy (2025), Attallah (2025). Histopathological examination of hematoxylin-and-eosin-stained whole-slide images (WSIs) remains the clinical gold standard for diagnosis, offering critical insights into tumor morphology, architectural organization, and cellular atypia. However, manual interpretation of gigapixel-scale WSIs is labor-intensive, inherently subjective, and susceptible to inter-observer variability—challenges further amplified by the pronounced tissue heterogeneity, multi-scale morphological diversity ranging from subcellular nuclear pleomorphism to glandular disarray, and subtle histological signatures of early neoplasia that characterize CRC progression. While deep learning has emerged as a powerful tool for computational pathology, conventional convolutional neural networks (CNNs) Anwar et al. (2018), Xu et al. (2019), Alzubaidi et al. (2021) are fundamentally constrained by their local receptive fields, limiting their capacity to model long-range spatial dependencies essential for contextualizing focal dysplastic changes within broader tissue architecture. Vision Transformers Dosovitskiy (2020), Liu et al. (2021), Wang et al. (2021), Wu et al. (2025) alleviate this limitation through global self-attention but introduce prohibitive quadratic computational complexity and often overlook diagnostically rich frequency-domain cues such as orientation-specific textures, edge sharpness, and structural regularity that are highly informative yet frequently suppressed in purely spatial pipelines. As illustrated in Figure 1, colorectal cancer diagnosis demands precise discrimination among visually similar yet clinically distinct entities: normal mucosa, serrated polyps (often precursors), adenomas (benign neoplasms), and invasive adenocarcinomas. The challenge lies in detecting subtle, localized deviations from mild nuclear atypia in early adenomas to complex glandular disarray in poorly differentiated carcinomas, that are easily overlooked by models relying solely on global features. Recent studies have highlighted the value of wavelet-based decomposition in medical imaging, where fixed transforms like Haar wavelets Haar (1909) provide an interpretable, shift-sensitive prior for disentangling multi-resolution features: low-frequency components capture coarse tissue layout, while high-frequency subbands explicitly encode horizontal, vertical, and diagonal edges corresponding to biologically meaningful structures including crypt alignment, stromal invasion, and nuclear membranes. Concurrently, state-space models such as Mamba Gu and Dao (2023), Ma et al. (2024), Ruan and Xiang (2024), Liu J. et al. (2024) offer a promising alternative to attention mechanisms by enabling selective, data-dependent propagation of information across long sequences with linear complexity, which is ideal for fusing hierarchical features from multi-scale pathology representations. Motivated by these complementary advances, we propose CRC-Former, a novel hybrid architecture that synergistically integrates frequency-aware representation learning and efficient cross-scale sequence modeling. Specifically, CRC-Former introduces two key innovations: first, a Frequency-aware Global-Local Transformer Block (FGT), which decomposes intermediate features via Haar wavelet transform and applies orientation-adaptive sliding-window attention within distinct subbands to enhance sensitivity to multi-directional pathological textures; and second, a Cross-Scale Mamba Block (CSM), which leverages selective state-space dynamics to fuse features across all spatial resolutions in a context-aware and computationally efficient manner. Together, these modules enable CRC-Former to simultaneously capture fine-grained cytoarchitectural anomalies and global tissue-level abnormalities, thereby addressing the core challenges of robust and scalable CRC classification in digital pathology.

## 2 Related work

Convolutional neural networks (CNNs) have historically formed the backbone of computer-aided diagnosis (CAD) systems in medical imaging Masood et al. (2020). Architectures such as ResNet He et al. (2016) and its many derivatives have been widely adopted for tasks ranging from anatomical structure classification to pathological lesion identification Cheema et al. (2019). Their strength lies in hierarchical feature learning and efficient extraction of local spatial patterns. Nevertheless, CNNs are inherently constrained by their localized receptive fields, limiting their ability to model long-range contextual interactions or exploit frequency-domain characteristics. To address these limitations, Vision Transformers (ViTs) Dosovitskiy (2020) introduced a paradigm shift by leveraging self-attention mechanisms to capture global dependencies across image patches. Subsequent variants like the Swin Transformer Liu et al. (2021) further enhanced practicality through hierarchical feature representation and localized window-based attention, balancing global context with computational feasibility. However, their self-attention operation incurs quadratic computational complexity with respect to sequence length. Moreover, conventional ViT architectures predominantly operate in the spatial domain and do not explicitly incorporate or leverage frequency-domain information, which could offer complementary cues to improve both model robustness and interpretability. More recently, State Space Models (SSMs) have emerged as a scalable alternative for modeling long-range dependencies with linear computational complexity Gu et al. (2021), Gu and Dao (2023). Building on this foundation, frameworks such as Mamba and their vision-specific adaptations have demonstrated competitive performance as visual backbones Liu J. et al. (2024), Liu Y. et al. (2024). These models efficiently integrate global contextual information while maintaining favorable computational properties, making them particularly well-suited for large-scale, high-resolution medical imaging tasks. Their capacity to jointly achieve efficiency, expressiveness, and scalability positions SSM-based architectures as promising candidates for advancing histopathology image classification.

## 3 Materials and methods

### 3.1 Datasets

We evaluate our method on the Chaoyang Dataset Zhu et al. (2021), a large-scale collection of whole-slide images (WSIs) retrospectively curated from routine clinical practice at Beijing Chaoyang Hospital. Representative non-overlapping patches of size 512×512 pixels were extracted from diagnostically relevant regions under the supervision of board-certified pathologists. The dataset comprises four clinically significant classes: Normal mucosa, Serrated lesions (including hyperplastic polyps and sessile serrated adenomas), Adenoma (tubular/tubulovillous), and Adenocarcinoma. In total, 6,160 annotated patches are included, distributed as follows: 1,816 Normal, 1,163 Serrated, 2,244 Adenocarcinoma, and 937 Adenoma. To ensure fair comparison, we adopt the consistent train and test partition: the training set contains 1,111 Normal, 842 Serrated, 1,404 Adenocarcinoma, and 664 Adenoma samples (total = 4,021); the test set includes 705 Normal, 321 Serrated, 840 Adenocarcinoma, and 273 Adenoma samples (total = 2,139). No overlap exists between training and test slides at the patient level, thereby mitigating data leakage and enabling assessment of generalization to unseen individuals. All experiments are conducted on this standardized split. Figure 1 shows some samples of the Chaoyang dataset.

### 3.2 Preliminaries: haar wavelet transform

The Haar wavelet transform Haar (1909) provides a computationally efficient, orthogonal multiresolution decomposition that is particularly well-suited for capturing localized intensity discontinuities—such as cell boundaries, nuclear membranes, and glandular edges—common in histopathological images. Given a 1D discrete signal $s\in R^{2^{J}}$ (with J∈N ), the Haar transform recursively computes approximation (scaling) coefficients $a_{j}\left[n\right]$ and detail (wavelet) coefficients $d_{j}\left[n\right]$ at scale j=0,1,…,J−1 via Equations 1, 2:

$$a_{j}\left[n\right]=\frac{1}{\sqrt{2}}\left(a_{j+1}\left[2n\right]+a_{j+1}\left[2n+1\right]\right),$$ (1)

$$d_{j}\left[n\right]=\frac{1}{\sqrt{2}}\left(a_{j+1}\left[2n\right]-a_{j+1}\left[2n+1\right]\right),$$ (2)

where $a_{J}\left[n\right]=s\left[n\right]$ denotes the original signal, and $n=0,1,\ldots ,2^{j}-1$ . The inverse transform reconstructs s exactly from $\left\{a_{0}\left[0\right]\right\}\cup {\left\{d_{j}\left[n\right]\right\}}_{j=0}^{J-1}$ .

For 2D images $X\in R^{H\times W}$ (assuming H,W are powers of two for simplicity), the 2D Haar transform applies the 1D decomposition along rows and columns successively. At each level, it yields four subbands {A,H,V,D} , where A is the low-frequency approximation (capturing coarse tissue architecture), while H , V , and D represent horizontal, vertical, and diagonal detail coefficients, respectively—encoding fine-scale texture, edge orientation, and structural irregularities. This explicit separation of spatial frequencies enables pathology-aware feature disentanglement: for instance, dysplastic nuclei often manifest as high-magnitude responses in diagonal/high-frequency bands, whereas tumor-stroma interfaces are reflected in vertical/horizontal edges. Owing to its simplicity, invertibility, and sensitivity to abrupt intensity changes, the Haar wavelet serves as an effective prior for modeling the multi-scale heterogeneity inherent in colorectal cancer histology.

### 3.3 Overview of CRC-Former

**FIGURE 2:**
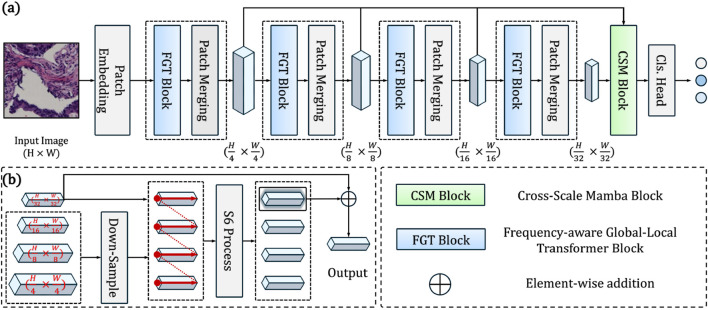
**(a)** Overview of the proposed CRC-Former: a hierarchical architecture with four Frequency-aware Global-Local Transformer (FGT) blocks and a Cross-Scale Mamba (CSM) block for global aggregation. **(b)** CSM block detail: fuses multi-scale features via downsampling, S6 processing, and element-wise addition to the finest-scale output.

We present CRC-Former, a hierarchical deep architecture designed for whole-slide image (WSI) classification in colorectal cancer pathology. As illustrated in Figure 2a, the network processes an input histopathology image $X\in R^{H\times W\times 3}$ through a multi-stage pipeline that progressively extracts and refines multi-scale, frequency-aware contextual features. The pipeline begins with a Patch Embedding layer that partitions X into non-overlapping patches of size P×P , followed by linear projection to obtain initial patch embeddings. These are then processed by a sequence of four Frequency-aware Global–Local Transformer Blocks (FGT Blocks), each operating at successively coarser spatial resolutions ( H/4×W/4 , H/8×W/8 , H/16×W/16 , H/32×W/32 ). Between consecutive FGT blocks, a Patch Merging operation reduces spatial dimensionality while doubling the channel depth—enabling efficient hierarchical feature abstraction. Each FGT block integrates Haar wavelet decomposition to decompose local patch representations into multi-frequency subbands, which are then fused via hybrid attention mechanisms to capture both global context and orientation-sensitive texture cues. After the final FGT stage, the high-level, low-resolution feature map is fed into a Cross-Scale Mamba Block (CSM Block), which serves as a global aggregator. Unlike conventional transformers or CNNs, the CSM Block leverages selective state-space modeling to dynamically fuse information across all previous resolution levels—effectively integrating fine-grained textural details from early stages with coarse semantic patterns from deeper layers. This enables the model to reason about long-range tissue organization while preserving discriminative local morphological signals. Finally, a global average pooling operation is applied, followed by a fully connected Classification Head to produce the predicted class probabilities. The entire architecture is end-to-end trainable and maintains linear computational complexity with respect to sequence length, making it scalable to gigapixel WSIs without sacrificing representational power.

### 3.4 Cross-Scale Mamba Block

The Cross-Scale Mamba Block (CSM, Figure 2b) serves as the global aggregation module of CRC-Former, designed to fuse multi-resolution features extracted by the preceding four FGT stages into a unified, context-aware representation. Formally, let $\left\{F_{1},F_{2},F_{3},F_{4}\right\}$ denote the feature maps output by the FGT blocks at resolutions $\left(\frac{H}{4}\times \frac{W}{4}\right)$ , $\left(\frac{H}{8}\times \frac{W}{8}\right)$ , $\left(\frac{H}{16}\times \frac{W}{16}\right)$ , and $\left(\frac{H}{32}\times \frac{W}{32}\right)$ , respectively, where each $F_{i}\in R^{H_{i}\times W_{i}\times C_{i}}$ . To enable cross-scale interaction, all four feature maps are first downsampled via bilinear interpolation (or strided convolution) to match the coarsest spatial resolution $\left(\frac{H}{32}\times \frac{W}{32}\right)$ , yielding (Equation 3):

$${\tilde{F}}_{i}=\text{DownSample}\left(F_{i}\right),\;i=1,2,3,4,$$ (3)

where ${\tilde{F}}_{i}\in R^{\frac{H}{32}\times \frac{W}{32}\times C_{i}}$ . These aligned representations are then concatenated along the channel dimension, which is given by Equation 4:

$$F_{\text{cat}}=\left[{\tilde{F}}_{1}\|{\tilde{F}}_{2}\|{\tilde{F}}_{3}\|{\tilde{F}}_{4}\right]\in R^{\frac{H}{32}\times \frac{W}{32}\times \overset{4}{\underset{i=1}{\sum }}C_{i}}.$$ (4)

The concatenated tensor $F_{\text{cat}}$ is reshaped into a sequence $z\in R^{L\times D}$ , where $L=\frac{H}{32}\cdot \frac{W}{32}$ denotes the number of spatial locations, and $D=\sum_{i=1}^{4}C_{i}$ is the total channel depth. This sequence is then processed by a Bidirectional S6 Layer (Bi-Mamba), which applies two parallel selective state-space models—one forward and one backward—to capture long-range dependencies in both spatial directions. The output sequence $z^{\prime }\in R^{L\times D}$ is reshaped back into a 3D tensor $F_{\text{mamba}}\in R^{\frac{H}{32}\times \frac{W}{32}\times D}$ .

To preserve the identity of the finest-scale representation while incorporating cross-scale context, we extract from $F_{\text{mamba}}$ only the channels corresponding to the original $F_{4}$ (i.e., the last $C_{4}$ channels), denoted as $F_{\text{mamba}}^{\left(4\right)}\in R^{\frac{H}{32}\times \frac{W}{32}\times C_{4}}$ . This is added element-wise to the original $F_{4}$ to form the final output (Equation 5):

$$F_{\text{out}}=F_{4}+F_{\text{mamba}}^{\left(4\right)}.$$ (5)

This design ensures that the CSM block enhances the discriminative power of the coarsest-scale features through globally aware, cross-resolution context modeling—without disrupting the hierarchical structure or introducing excessive computational overhead. The use of Bi-Mamba enables efficient, linear-complexity aggregation across all scales, making the CSM block particularly suitable for histopathology analysis.

### 3.5 Frequency-aware Global-Local Transformer Block

**FIGURE 3:**
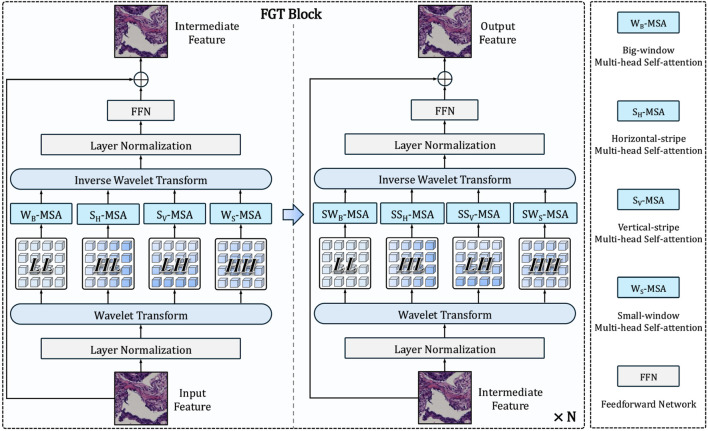
Architecture of the proposed Frequency-aware Global-Local Transformer (FGT) block. It decomposes input features via Haar wavelet transform, applies orientation- and scale-specific multi-head self-attention (e.g., $W_{B}$ -MSA for global context, $S_{H}$ -MSA for horizontal textures), then reconstructs fused features via inverse transform.

The Frequency-aware Global-Local Transformer Block (FGT, Figure 3) is the core building block of CRC-Former’s backbone, designed to jointly model global contextual dependencies and multi-directional local texture patterns via Haar wavelet-guided attention. As depicted in Figure 3, each FGT block processes an input feature map $F_{\text{in}}\in R^{H^{\prime }\times W^{\prime }\times C}$ at a fixed spatial resolution $\left(H^{\prime },W^{\prime }\right)$ and produces an output $F_{\text{out}}\in R^{H^{\prime }\times W^{\prime }\times C}$ through a hierarchical frequency decomposition and fusion pipeline.

**FIGURE 4:**
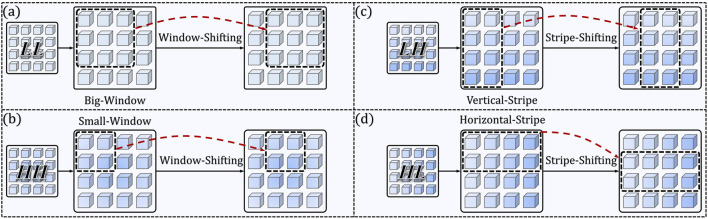
Detailed illustration of the four specialized MSA variants. The Window-Shifting and Stripe-Shifting operations are visualized via red dashed arrows, demonstrating how shifted windows/stripes enable cross-window communication, which is critical for maintaining connectivity across partitioned regions without introducing extra parameters. **(a)** WB-MSA. **(b)** WS-MSA. **(c)** SV-MSA. **(a)** SH-MSA.

First, the input feature map undergoes Layer Normalization, followed by a Haar Wavelet Transform that decomposes it into four orthogonal subbands, which is given by Equation 6:

$$\left\{F_{LL},\;F_{HL},\;F_{LH},\;F_{HH}\right\}=\text{WT}\left(F_{\text{in}}\right),$$ (6)

where $F_{LL}\in R^{\frac{H^{\prime }}{2}\times \frac{W^{\prime }}{2}\times C}$ represents the low-frequency approximation (coarse tissue structure), while $F_{HL},F_{LH},F_{HH}\in R^{\frac{H^{\prime }}{2}\times \frac{W^{\prime }}{2}\times C}$ encode horizontal, vertical, and diagonal high-frequency details (e.g., glandular edges, nuclear borders), respectively. Each subband is then processed independently by a dedicated multi-head self-attention (MSA) module with spatially constrained windowing (Figure 4):

-
$W_{B}$ -MSA: Applies big-window MSA over the entire $F_{LL}$ to capture long-range global context;
-
$S_{H}$ -MSA: Applies horizontal-stripe MSA along rows of $F_{HL}$ to enhance sensitivity to horizontally oriented textures;
-
$S_{V}$ -MSA: Applies vertical-stripe MSA along columns of $F_{LH}$ to emphasize vertically oriented structures;
-
$W_{S}$ -MSA: Applies small-window MSA over $F_{HH}$ to preserve fine-grained, localized textural anomalies.

These frequency-specific attention outputs are concatenated and passed through an Inverse Wavelet Transform to reconstruct a fused feature map $F_{\text{fused}}\in R^{H^{\prime }\times W^{\prime }\times C}$ . A residual connection from the original $F_{\text{in}}$ is added before applying Layer Normalization and a Feedforward Network (FFN), yielding the final output of the process (Equation 7):

$$F_{\text{out}}=\text{FFN}\left(\text{LN}\left(F_{\text{fused}}+F_{\text{in}}\right)\right).$$ (7)

To further enhance representational capacity, the FGT block is stacked N times within each stage, enabling progressive refinement of both global architecture and local texture cues. The use of sliding-window (denoted by prefix “S”) ensures efficient computation while preserving orientation-sensitive discriminative power, critical for distinguishing subtle histopathological phenotypes in colorectal cancer.

## 4 Results

### 4.1 Implementation details

To ensure a fair and reproducible comparison with prior works Liu S. et al. (2024), we strictly adhere to the same training protocol throughout our experiments. Input patches are resized to 224×224 pixels, a standard resolution widely used in vision-based histopathology analysis. During preprocessing, we apply only minimal augmentation: random horizontal flipping, and channel-wise normalization using ImageNet-derived statistics ( μ=[0.485,0.456,0.406] , σ=[0.229,0.224,0.225] ) Deng et al. (2009). No advanced augmentation strategies are employed, thereby isolating architectural improvements from data manipulation effects. The model is optimized using the Adam optimizer Adam (2014) with an initial learning rate of $1\times 10^{-4}$ . The learning rate is decayed over time via cosine annealing without restarts, where the period parameter $T_{\text{max}}$ is set to 10 epochs. Training proceeds for a fixed budget of 300 epochs with a batch size of 32, striking a balance between memory constraints and gradient stability. All experiments are implemented in PyTorch Paszke et al. (2019) and executed on a single NVIDIA A100 GPU with 40 GB of memory. We report results averaged over three independent runs with different random seeds to account for stochastic variability.

### 4.2 Evaluation metrics

To ensure a comprehensive and robust assessment, we employ multiple complementary evaluation metrics. Specifically, we report four standard classification metrics: Accuracy (Acc) Powers (2020), AUC Bradley (1997), Precision, Recall Baeza-Yates et al. (1999), and F1 score Sokolova and Lapalme (2009). Accuracy reflects the overall proportion of correctly classified samples and remains a fundamental indicator in diagnostic tasks. Precision and Recall, widely used in medical AI, assess different aspects of predictive quality: Precision measures the fraction of true positive predictions among all samples predicted as positive, while Recall quantifies the model’s ability to identify all actual positive cases. Since these two metrics often exhibit a trade-off—improving one may degrade the other—we further adopt the F1 score, defined as their harmonic mean, to provide a balanced evaluation of classification performance. In addition, we evaluate model discriminability using the receiver operating characteristic (ROC) curve and the corresponding area under the curve (AUC), which offer threshold-invariant measures of diagnostic capability across all classes. These evaluation metrics are summarized as the following Equations 8–12:

$$\text{Accuracy}\left(\text{ACC}\right)=\frac{\text{TP}+\text{TN}}{\text{TP}+\text{FP}+\text{TN}+\text{FN}},$$ (8)

$$\text{Precision}=\frac{\text{TP}}{\text{TP}+\text{FP}},$$ (9)

$$\text{Recall}=\frac{\text{TP}}{\text{TP}+\text{FN}}.$$ (10)

$$\text{F}1\text{ Score}=\frac{2\cdot \text{Precision}\cdot \text{Recall}}{\text{Precision}+\text{Recall}}.$$ (11)

$$\text{AUC}=\frac{\sum_{i\in \text{positiveClass}}{\text{rank}}_{i}-M\left(1+M\right)/2}{M\cdot N},$$ (12)

where M is the number of positive samples, N is the number of negative samples, and ${\text{rank}}_{i}$ denotes the rank of sample i based on the model’s predicted probability (sorted in descending order).

### 4.3 Experimental results and analysis

Table 1 presents a comprehensive comparison of our proposed CRC-Former against the representative architectures including ResNet101 He et al. (2016), EfficientNet Tan and Le (2019), ViT-B Dosovitskiy (2020), Swin-S Liu et al. (2021), ConvNext Liu et al. (2022), InceptionNext Yu et al. (2024), TransXNet Lou et al. (2025), BiFormer Zhu et al. (2023), GroupMixFormer Ge et al. (2023), Eff-CTM Liu S. et al. (2024), MedMamba Yue and Li (2024) and SBTAYLOR-KAN Fatema et al. (2025), across five standard classification metrics: Accuracy, F1 score, Precision, Recall, and AUC. As shown, CRC-Former achieves state-of-the-art performance on the Chaoyang colorectal histopathology dataset, outperforming all baseline models in every metric.

**TABLE 1:** Quantitative performance comparison results with ten classic SOTA methods on the Chaoyang dataset.

Model	Year	Accuracy(%) ↑	F1(%) ↑	Precision(%) ↑	Recall(%) ↑	AUC(%) ↑
ResNet101	2016	83.92	77.32	79.61	76.48	85.46
EfficientNet-B0	2019	84.71	79.57	80.33	78.94	86.86
ViT-B	2021	81.53	76.02	77.59	74.94	84.22
Swin-S	2021	85.13	80.29	81.09	78.93	87.44
ConvNext-S	2022	78.35	71.89	71.55	72.42	82.62
InceptionNext	2023	84.81	79.70	80.23	79.25	87.05
TransXNet	2023	84.53	79.17	80.20	78.43	86.60
BiFormer	2023	83.12	76.28	78.60	75.91	85.03
GroupMixFormer	2023	85.09	79.78	80.65	79.24	87.07
Eff-CTM	2024	86.30	81.87	81.69	82.16	88.82
MedMamba	2024	85.01	79.67	80.59	79.20	86.99
SBTAYLOR-KAN	2025	84.62	79.22	80.15	78.83	86.53
Ours (CRC-Former)	– –	**87.42**	**83.11**	**82.84**	**83.33**	**89.67**

The best results are highlighted in bold.

Specifically, CRC-Former attains an accuracy of 87.42%, surpassing the previous best (Eff-CTM, 86.30%) by +1.12 percentage points. More importantly, it demonstrates superior discriminative power through its balanced high scores across all metrics: 83.11% F1 score, 82.84% precision, 83.33% recall, and 89.67% AUC. Notably, the model’s high recall (83.33%) indicates strong sensitivity to malignant tissue regions—including early adenomas and poorly differentiated carcinomas—while its precision (82.84%) reflects reliable suppression of false positives (e.g., misclassifying inflamed or hyperplastic mucosa as cancer). The macro-averaged F1 score further confirms robustness across all four diagnostic classes, critical for real-world deployment where class imbalance is common. Compared to recent transformer-based approaches such as BiFormer (83.12% Acc) and GroupMixFormer (85.09% Acc), CRC-Former’s consistent gains suggest that its hybrid design—integrating frequency-aware attention and cross-scale state-space modeling—is particularly effective at capturing the multi-scale, heterogeneous texture patterns characteristic of colorectal neoplasia. Unlike CNNs or pure transformers, which often prioritize either local edges or global context, CRC-Former explicitly decomposes tissue morphology into interpretable frequency subbands via Haar wavelets, then fuses them adaptively using Mamba’s selective state-space mechanism. This enables the model to simultaneously resolve fine nuclear atypia (high-frequency bands) and glandular architectural distortion (low-frequency bands)—features that are often missed by conventional architectures.

**FIGURE 5:**
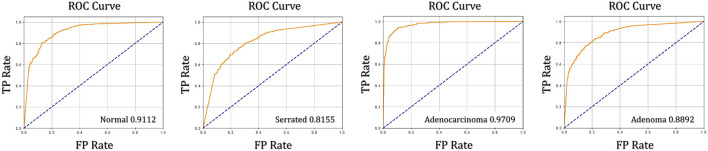
ROC curves and AUC values of the four different classes in the dataset. CRC-Former achieves strong performance across all four classes: Normal (AUC = 0.9112), Serrated lesion (AUC = 0.8155), Adenoma (AUC = 0.8892), and Adenocarcinoma (AUC = 0.9709).

Moreover, the elevated 89.67% average AUC score (the ROC curves and AUC scores of the four specific classes in the dataset are shown in Figure 5) underscores the model’s ability to maintain high discriminative power across varying decision thresholds—a key requirement for clinical screening systems aiming to minimize both false negatives (missed cancers) and false positives (unnecessary biopsies). When viewed holistically, CRC-Former’s dominance across all metrics validates its capacity to generalize beyond simple patch-level classification: it learns clinically meaningful representations that align with pathologist reasoning—balancing specificity, sensitivity, and interpretability.

In summary, these results demonstrate that CRC-Former not only advances the state of the art in colorectal cancer classification but also offers a scalable, architecture-driven solution tailored to the unique challenges of gigapixel histopathology. Its integration of signal processing priors with modern sequence modeling opens new avenues for developing robust, efficient, and explainable AI tools for digital pathology.

### 4.4 Ablation study

To validate the effectiveness of our architectural components—and specifically to assess whether attention in the frequency domain offers advantages over conventional spatial-domain sliding-window mechanisms—we conduct an ablation study using the standard Swin Transformer Small (Swin-S) Liu et al. (2021) as the baseline. Swin-S employs spatially localized, window-based self-attention and serves as a strong representative of current state-of-the-art hierarchical vision backbones.

As shown in Table 2, the Swin-S baseline achieves 85.13% accuracy and 87.44% AUC on the Chaoyang dataset. Replacing its spatial sliding-window attention with our Frequency-aware Global–Local Transformer Block (FGT)—which applies orientation-specific sliding windows ( $S_{H}$ , $S_{V}$ ) and multi-scale windows ( $W_{B}$ , $W_{S}$ ) in the Haar wavelet subbands—yields a notable improvement to 86.69% accuracy and 89.01% AUC (Model $B_{2}$ ). This gain demonstrates that decomposing features into frequency subbands and applying structure-aware attention within each band enables more discriminative modeling of histopathological textures (e.g., horizontal crypt alignment in $F_{HL}$ , vertical stromal invasion in $F_{LH}$ ) than uniform spatial windows. Separately, augmenting the Swin-S backbone with the Cross-Scale Mamba Block (CSM)—which fuses features from all four stages via selective state-space modeling—improves performance to 86.05% accuracy and 88.62% AUC (Model $B_{1}$ ), confirming the value of efficient, long-range cross-resolution context aggregation. The full CRC-Former, integrating both FGT and CSM, achieves the best results: 87.42% accuracy, 83.11% F1 score, and 89.67% AUC. Crucially, the consistent superiority of FGT over Swin-S provides direct evidence that frequency-domain sliding-window attention is more effective than its spatial counterpart for capturing the multi-orientation, multi-scale morphological signatures of colorectal neoplasia. The complementary gains from CSM further indicate that enhanced local representation must be coupled with global cross-scale reasoning to maximize diagnostic performance. This ablation study not only quantifies the contribution of each module but also establishes a key insight: leveraging wavelet-based frequency decomposition as an inductive bias for attention design leads to more pathology-aware feature learning than purely spatial mechanisms—a finding with broader implications for vision transformers in the field of medical image analysis.

**TABLE 2:** Ablation study of the proposed FGT and CSM modules in CRC-Former on the Chaoyang dataset.

Model	Swin	FGT	CSM	Accuracy(%) ↑	F1(%) ↑	Precision(%) ↑	Recall(%) ↑	AUC(%) ↑
Baseline	✓	✗	✗	85.13	80.29	81.09	79.83	87.44
$B_{1}$	✓	✗	✓	86.05	80.96	81.91	80.91	88.62
$B_{2}$	✓	✓	✗	86.69	81.70	81.56	82.47	89.01
CRC-Former	✓	✓	✓	**87.42**	**83.11**	**82.84**	**83.33**	**89.67**

The best results are highlighted in bold.

## 5 Discussion

CRC-Former holds significant clinical potential as an AI-powered decision support tool in colorectal cancer pathology: by delivering rapid, accurate, and interpretable classification of whole-slide images—including critical precancerous (adenoma, serrated) and malignant (adenocarcinoma) lesions—it can assist pathologists in reducing diagnostic variability, accelerating turnaround time, and improving early detection rates, particularly in settings with limited expert resources. Its consistent performance across lesion types and compatibility with digital pathology workflows position it as a scalable solution for standardizing CRC diagnosis and enhancing screening quality in real-world clinical practice. Our results demonstrate that CRC-Former achieves state-of-the-art performance in colorectal cancer classification on the Chaoyang histopathology dataset, significantly outperforming both CNN- and transformer-based baselines across all evaluation metrics. This success stems from a deliberate architectural shift: rather than treating histopathology images as generic visual data, we embed domain-specific priors—namely, multi-scale texture heterogeneity and frequency-domain discriminability—directly into the model’s inductive bias. The integration of Haar wavelet decomposition with orientation-aware sliding-window attention (FGT) enables the model to resolve diagnostically critical patterns—such as crypt distortion, nuclear pleomorphism, and stromal invasion—that are often lost in spatial-only representations. Meanwhile, the Cross-Scale Mamba Block (CSM) provides an efficient mechanism for long-range contextual reasoning without the quadratic overhead of self-attention, making the architecture scalable to high-resolution pathology workflows. Notably, the ablation study provides compelling evidence that frequency-domain modeling is not merely an auxiliary enhancement but a core enabler of performance gains. The consistent superiority of FGT over Swin-S—a strong spatial baseline—validates our hypothesis that pathological textures are better characterized in spectral subbands than in raw pixel space. This insight challenges the prevailing paradigm in medical vision transformers, which largely operate in the spatial domain, and suggests that hybrid signal-processing–deep-learning approaches may offer a more principled path toward clinically robust AI. From a clinical perspective, CRC-Former’s balanced precision and recall (82.84% and 83.33%, respectively) indicate low rates of both false positives and false negatives—critical for minimizing unnecessary biopsies and missed cancers. In summary, CRC-Former exemplifies a new design philosophy for computational pathology: one that unifies classical signal analysis with modern sequence modeling to build systems that are not only accurate but also efficient, interpretable, and aligned with medical domain knowledge.

## 6 Conclusion

We propose CRC-Former, a frequency-aware architecture for colorectal cancer classification in histopathology images. Departing from spatial-only attention, CRC-Former integrates Haar wavelet–based multi-scale spectral attention (FGT block) and selective state-space modeling for cross-resolution fusion (CSM block). On the Chaoyang dataset, it achieves state-of-the-art accuracy (87.42%) and AUC (89.67%), outperforming CNNs, ViTs, and hybrids. Ablations confirm the benefit of frequency-domain modeling, demonstrating the value of wavelet-based inductive bias in medical vision. This work bridges signal processing and deep learning for more efficient, interpretable pathology AI.

## Data Availability

The original contributions presented in the study are included in the article/supplementary material, further inquiries can be directed to the corresponding author.
